# Casein hydrolysate promotes intestinal repair in adult mice with *Salmonella enteritis* through a lactate-GPR81-Wnt3A axis in intestinal stem cell niche

**DOI:** 10.3389/fimmu.2026.1817158

**Published:** 2026-06-15

**Authors:** Jianbo Cheng, Jing Li, Yuying Bai, Shi Wang, Lirui Sun

**Affiliations:** 1 Key Laboratory for Food Science & Engineering, Harbin University of Commerce, Harbin, China; 2 Heilongjiang Feihe Dairy Co., Ltd, Qiqihar, China

**Keywords:** Casein hydrolysate, gut microbiota, intestinal stem cells, lactate, Paneth cell, *S. Typhimurium*

## Abstract

**Background:**

Salmonella enterica serovar Typhimurium (S. Typhimurium) is a major enteric pathogen that causes severe intestinal damage, characterized by epithelial disruption, inflammation, and impaired regeneration. Nutritional intervention has emerged as a promising strategy to mitigate such injury and promote mucosal repair. Casein enzymatic hydrolysate (CEH) possesses well-documented anti-inflammatory and mucosa-protective properties. However, whether CEH exerts its protective effects against S. Typhimurium-induced intestinal injury by modulating the gut microbiota, and the underlying mechanisms, remains largely unknown.

**Methods:**

Twelve−month−old C57BL/6J mice were pretreated with streptomycin and challenged with S. Typhimurium. In the primary efficacy study, mice were assigned to four groups: control, control + CEH, model, and model + CEH (2% CEH in diet for 10 days before and throughout infection). Disease severity, intestinal histology (H&E and PAS staining), inflammation (TNF−α and IL−10 ELISA; CD45^+^ flow cytometry), barrier function (E−cadherin flow cytometry; PEPT1/SGLT1 immunofluorescence), stem cell activity (LGR5/Ki67 and CD24^+^LGR5^+^ staining), Paneth cell niche factors (LYZ1/Wnt3A immunofluorescence and western blot), gut microbiota composition (16S rDNA sequencing), and luminal L−lactate levels (biochemical assay) were evaluated. To establish causality, additional mechanistic experiments were performed using exogenous L−lactate supplementation, the GPR81 inhibitor 2,5−DHBA, and the Wnt secretion inhibitor Wnt−C59, with the same panel of tests.

**Results:**

CEH administration significantly improved survival, alleviated clinical disease severity, and attenuated systemic and intestinal inflammation. CEH preserved mucosal architecture, restored goblet cells and barrier proteins (PEPT1, SGLT1, E−cadherin), and promoted LGR5^+^ and CD24^+^LGR5^+^ intestinal stem cell regeneration. CEH reshaped the gut microbiota, enriched lactate−producing genera, and increased luminal L−lactate levels, accompanied by restored Paneth cell LYZ1 expression and elevated Wnt3A in the crypt niche. Strikingly, lactate supplementation improved CEH’s protective effects on Paneth cell function, ISC proliferation, barrier integrity, and inflammatory cytokines. LYZ1^+^ Paneth cells co−expressed the lactate−sensing receptor GPR81. Pharmacological blockade of GPR81 or Wnt abrogated the beneficial effects of both CEH and lactate, confirming that CEH acts through the lactate−GPR81−Wnt3A axis.

**Conclusion:**

Collectively, these findings demonstrate that CEH alleviates S. Typhimurium-induced enteritis in adult mice via a microbiota-lactate-GPR81-Wnt3A-ISC axis. CEH represents a promising nutritional strategy to counteract infection-induced intestinal injury and promote regeneration in adult hosts with diminished regenerative reserve.

## 1 Introduction

Intestinal frailty resulting from prior infections, antibiotic exposure, or dietary factors can increase susceptibility to enteritis induced by Salmonella enterica serovar Typhimurium (S. Typhimurium). Impaired intestinal barrier integrity, dysregulated inflammatory responses, and diminished regenerative capacity of the epithelium are key features that predispose hosts to severe infection and delayed recovery (1, 2). Although Salmonella-induced enteritis is a major foodborne disease worldwide, effective host-directed nutritional interventions that promote mucosal repair remain scarce (3–5). Therefore, there is an urgent need for safe, nutrition−based strategies to enhance intestinal resilience and accelerate epithelial regeneration following bacterial infection (6, 7).

The intestinal epithelium is maintained by a pool of rapidly cycling intestinal stem cells (ISCs), located at the crypt base and marked by LGR5(leucine-rich repeat-containing G-protein-coupled receptor 5) expression. These ISCs drive continuous epithelial renewal and are essential for restoring tissue integrity after injury (8). Their function critically depends on a specialized niche in which Paneth cells play a central role. Paneth cells reside adjacent to ISCs and provide key trophic and signaling inputs, including the secretion of Wnt ligands such as Wingless-type mouse mammary tumor virus integration site family member 3A (Wnt3A), antimicrobial peptides such as lysozyme (LYZ1), and other niche factors (9). Through these signals, Paneth cells sustain ISC self-renewal, proliferation, and lineage commitment, thereby maintaining villus–crypt architecture and barrier function (10). However, enteric pathogens, including Salmonella, can disrupt this niche (11). Infection impairs Paneth cell antimicrobial activity and down-regulates Wnt signaling, leading to ISC depletion, villus atrophy, crypt shortening, and persistent epithelial defects (12, 13). In adult hosts, such pathogen-induced perturbations are superimposed on pre-existing declines in Paneth cell function and ISC fitness, resulting in more severe tissue damage and delayed repair.

The gut microbiota has emerged as a crucial regulator of intestinal homeostasis and regeneration (14). Dysbiosis induced by infection, aging, or antibiotics is frequently associated with impaired barrier integrity and chronic inflammation (15). Among commensal microbes, lactate–producing bacteria (lactate acid bacteria, LAB) are of particular interest. Genera such as Ligilactobacillus, Streptococcus, Enterococcus, and Lactococcus produce substantial amounts of lactate, which acts not only as a metabolic substrate but also as a signaling molecule within the intestinal ecosystem (16). L-lactate can engage lactate-sensing G protein–coupled receptors (e.g., GPR81/HCAR1) on intestinal epithelial and immune cells, thereby modulating inflammatory pathways, barrier proteins, and potentially niche cell function (17, 18). Notably, accumulating evidence has established a direct link between microbiota−derived lactate and ISC activity. Lee et al. demonstrated that lactate derived from LAB stimulates ISC proliferation through Wnt/β−catenin signals emanating from Paneth cells and intestinal stromal cells in a Gpr81−dependent manner (19, 20). More recently, Wu et al. reported that Lactobacillus amylovorus regulates jejunal stem cell function via the Lactobacillus−lactate−GPR81 axis, showing that lactate activates Wnt/β−catenin signaling in a GPR81−dependent manner to promote ISC−mediated epithelial proliferation (21). This emerging paradigm positions lactate as a key microbial metabolite that bridges gut microbiota composition with stem cell-driven epithelial regeneration. However, the specific involvement of the lactate−GPR81−Wnt axis in the context of Salmonella−induced enteritis in hosts, particularly whether nutritional interventions can harness this axis to counteract infection−induced intestinal injury, remains largely unexplored.

Dietary bioactive peptides represent a promising class of modulators of both host physiology and the microbiota. The Casein enzymatic hydrolysate (CEH) was generated by controlled proteolysis of milk casein, contains a complex mixture of peptides with reported anti-inflammatory, antioxidant, and mucosa-protective properties (17, 18, 22). It has also been proposed to act as a prebiotic-like substrate that may support the growth and metabolic activity of beneficial bacteria, including LAB (23–25). Several recent studies have identified specific casein−derived peptides with immunomodulatory and barrier−protective activities. For instance, the casein peptide SPAQILQW activates macrophages via the TLR2/TLR4−MAPK pathways (26), while the casein phosphopeptide SIER1P protects intestinal epithelial barrier integrity via the CaSR/NF−κB signaling pathway (27). However, whether CEH as a whole ingredient can protect the adult intestine during bacterial infection, and whether its protective effects are mediated through the microbiota−lactate−Paneth cell−Wnt−ISC axis, have not been systematically investigated.

Therefore, this study aimed to comprehensively evaluate the protective effects of dietary CEH supplementation against S. Typhimurium−induced enteritis in adult mice. We characterized associated changes in gut microbiota composition, luminal L−lactate levels, Paneth cell function, and ISC activity. Furthermore, we performed intervention experiments using exogenous lactate supplementation and pharmacological blockade of GPR81 and Wnt signaling to verify the mechanism of the protective effects of CEH. Our findings support that the CEH could be used as a functional nutritional intervention for intestinal protection against bacterial infection, particularly in adult populations with diminished regenerative reserve.

## 2 Materials and methods

### 2.1 Ethical approval

All animal experiments were approved by the Animal Ethics Committee of Harbin University of Commerce (approval number: HUCAEC-2024-003) and were conducted in strict accordance with the Guide for the Care and Use of Laboratory Animals. All efforts were made to minimize animal suffering, and experimenters were blinded to group allocation during data collection and analysis.

### 2.2 Animals

Male C57BL/6J mice (12-month-old, 15–35 g) were purchased from Beijing Vital River Laboratory Animal Technology Co., Ltd. All animals were acclimatized for one week under specific pathogen-free (SPF) conditions with a 12 h light/dark cycle, a controlled temperature of 22–24 °C, relative humidity of 28–50%, and ad libitum access to standard chow and water.

### 2.3 Bacterial strain

S. Typhimurium strain BNCC333565 was cultured at 37 °C for 18–24 h on nutrient broth (NB) agar plates or in NB liquid medium. Bacterial concentration was estimated by measuring the optical density at 600 nm (OD_600_) and further confirmed by serial dilution and plate counting on NB agar. For infection, bacteria were harvested by centrifugation, washed twice with sterile phosphate-buffered saline (PBS), and resuspended to a final concentration of 1.0 × 10^8^ colony-forming units (CFU)/mL in PBS.

### 2.4 Preparation of casein hydrolysate

Bovine casein was dispersed in deionized water at a final concentration of 5% (w/v). The pH of the suspension was adjusted to 8.0, and limited enzymatic hydrolysis was carried out at 55 °C using alkaline protease 2.4L (Novozymes, activity: 2.4 AU/g) at an enzyme-to-substrate mass ratio of 2% for 4 h. The reaction was terminated by heating at 90 °C for 15 min. The hydrolysate was cooled, centrifuged at 8,000 × g for 20 min at 4 °C, and the supernatant was collected and lyophilized to obtain the CEH. The peptide content of CEH was ≥85%, as determined by the Kjeldahl method.

### 2.5 Experimental design

After the adaptation period, mice were randomly assigned to nine groups (n = 6 per group):

Control: uninfected mice fed a standard diet.

Model: mice infected with S. Typhimurium and fed a standard diet.

Control + CEH: uninfected mice fed a standard diet supplemented with 2% (w/w) CEH.

Model + CEH: infected mice fed a standard diet supplemented with 2% (w/w) CEH starting 10 days before infection and maintained throughout the infection period.

Model + lactate: infected mice were supplemented with lactate throughout the infection period.

Model + lactate + 2,5-DHBA (2,5-Dihydroxybenzoic acid, Gentisic acid): infected mice were fed with lactate and intraperitoneally injected with the GPR81 inhibitor (2,5-DHBA, 15mM; HY-W001179, MedChemExperss) once a week.

Model + lactate + Wnt-C59: infected mice were fed lactate and administered the Wnt inhibitor Wnt-C59 (HY-15659, MedChemExpress) by oral gavage at 5 mg/kg once daily for 3 consecutive days.

Model + CEH + 2,5-DHBA: infected mice fed a standard diet supplemented with 2% (w/w) CEH starting 10 days before infection and maintained throughout the infection period and intraperitoneally injected with the 5-DHBA once a week.

Model + CEH + Wnt-C59: infected mice fed a standard diet supplemented with 2% (w/w) CEH starting 10 days before infection and administered the Wnt-C59 by oral gavage at 5 mg/kg once daily for 3 consecutive days.

To facilitate Salmonella colonization, all mice were orally administered streptomycin (20 mg per mouse in 100 μL PBS) 24 h before bacterial challenge to transiently disrupt the intestinal microbiota. Enteritis was induced by oral gavage of 2 × 10^8^ CFU S. Typhimurium in 200 μL PBS once daily for three consecutive days. Body weight and clinical symptoms, including stool consistency and presence of blood, were monitored daily. On day 7 post-infection, mice were euthanized, and serum, ileum, colon, and spleen tissues were collected for subsequent analyses.

### 2.6 Inclusion and exclusion criteria

Exclusion criteria were predefined before the experiment based on pilot studies. Mice were excluded from the primary analysis if either of the following occurred: i) No detectable Salmonella in feces at 48 h post-infection (detection limit: 10^2^ CFU/g), or ii)Fecal Salmonella load consistently < 10^4^ CFU/g at all three time points (days 2, 4, 6 post-infection).

Mice that died before day 2 post-infection were excluded from disease severity analysis but were included in survival analysis (as events).

To address potential selection bias, all mice (including excluded ones) were retained in sensitivity analyses using: i) Intention-to-treat-like censoring (non-responders censored at day 2 in survival analysis), and ii) Worst-case imputation for disease severity scores.

Experimenters were blinded to group allocation during data collection and analysis. Cage positions were rotated weekly.

### 2.7 Tissue processing

Ileum tissues were fixed in 4% paraformaldehyde at room temperature for 48 h, dehydrated through a graded ethanol series, cleared in xylene, and embedded in paraffin. Sections of 4 μm thickness were cut using a microtome (HistoCore MULTICUT, Leica, Germany).

### 2.8 H&E staining

Paraffin sections were deparaffinized, rehydrated, and stained with hematoxylin (C0107, Beyotime) and eosin (C0109, Beyotime) according to standard protocols. Sections were examined under a light microscope (BX53, Olympus). Villus height and crypt depth were measured using ImageJ software (National Institutes of Health, USA).

### 2.9 PAS staining

Goblet cells were visualized using a PAS staining kit (G1280, Solarbio) following the manufacturer’s instructions. PAS-positive goblet cells were counted in randomly selected 200× fields for each section.

### 2.10 Immunofluorescence staining

For antigen retrieval, sections were heated in citrate buffer (10 mM, pH 6.0). After cooling, sections were blocked with 1% bovine serum albumin (BSA; 4240GR100, BioFRoxx) for 1 h at room temperature and then incubated overnight at 4 °C with the following primary antibodies: rabbit anti-LGR5 (PA5-23000, Thermo Fisher, 1:100), mouse anti-Ki67 (14-5698-82, Thermo Fisher, 1:200), rabbit anti-PEPT1 (DF4266, Affinity, 1:500), rabbit anti-SGLT1 (GTX105367, GeneTex, 1:100), rabbit anti-Wnt3A (A25322, Abclonal, 1:200), rabbit anti-GPR81 (20146-1-AP, Proteintech, 1:300) and mouse anti-LYZ1 (518083, Santa Cruz Biotechnology, 1:50). After washing with PBS, sections were incubated with Alexa Fluor 488- or 594-conjugated secondary antibodies (Thermo Fisher, 1:500) for 1 h at room temperature in the dark. Nuclei were counterstained with DAPI (BS097, Biosharp, 1:1000). Fluorescence images were acquired using a BX53 fluorescence microscope (Olympus), and the percentage area of positive staining was quantified using ImageJ.

### 2.11 ELISA and biochemical assays

For cytokine and metabolite measurements, 0.1 g of intestinal tissue was homogenized in 0.9 mL ice-cold PBS and centrifuged at 2,500 × g for 10 min at 4 °C. The supernatant was collected for analysis. The concentrations of TNF-α and IL-10 were measured using commercial ELISA kits (TNF-α: ml002095; IL-10: ml037873; Shanghai Enzyme-Linked Biotechnology Co., Ltd.) according to the manufacturer’s instructions. L-lactate levels were determined using a biochemical assay kit (A019-2-1, Nanjing Jiancheng Bioengineering Institute) following the manufacturer’s protocol. Absorbance was measured at 450 nm using a microplate reader, and concentrations were calculated from the corresponding standard curves.

### 2.12 Western blot

Intestinal tissues were homogenized in ice−cold RIPA lysis buffer supplemented with protease inhibitors. After centrifugation at 13,000 × g for 10 min at 4 °C, supernatants were collected and quantified with a BCA kit. Equal protein was separated by 10% or 15% SDS−PAGE and transferred to PVDF membranes. Membranes were blocked with 5% non−fat milk in TBST for 2 h at room temperature, then incubated overnight at 4 °C with primary antibodies against Wnt3A (A25322, Abclonal, 1:700), LYZ1 (518083, Santa cruz, 1:1000), and β−actin (internal control). After washing, membranes were incubated with Goat Anti-Rabbit IgG Antibody, Peroxidase Conjugated (AP132P, Sigma, 1:10000) for 1 h at room temperature. Protein bands were detected by ECL and quantified using ImageJ; target protein levels were normalized to β−actin.

### 2.13 Flow cytometry

Single-cell suspensions were prepared from intestinal tissue by mechanical dissociation followed by enzymatic digestion with collagenase I (1 mg/mL) and DNase I (0.1 mg/mL) at 37 °C for 30 min. The cell suspension was passed through a 70 μm cell strainer, washed, and resuspended in PBS containing 2% fetal bovine serum. For surface staining, cells were incubated with fluorochrome-conjugated anti-CD45 (417-0451-80, Thermo Fisher, 1:40) and anti-E-cadherin (147303, BioLegend, 1:20) antibodies at 4 °C for 30 min in the dark. For intracellular LGR5 staining, cells were fixed and permeabilized according to standard procedures, then incubated with rabbit anti-LGR5 (PA5-23000, Thermo Fisher, 1:50), followed by an appropriate fluorophore-conjugated secondary antibody. Flow cytometric analysis was performed on a NovoCyte 3110 flow cytometer (Agilent), and data were analyzed using FlowJo software (v10.8, BD Biosciences).

### 2.14 16S rDNA gene sequencing of intestinal microbiota

Total genomic DNA from fecal samples was extracted using a fecal genomic DNA extraction kit (AU46111-96, BioTeke, China) according to the manufacturer’s instructions. DNA concentration was quantified using a Qubit fluorometer (Invitrogen, USA). The V3–V4 region of the bacterial 16S rRNA gene was amplified by PCR using universal primers 341F (5′-CCTACGGGNGGCWGCAG-3′) and 805R (5′-GACTACHVGGGTATCTAATCC-3′). PCR conditions were as follows: initial denaturation at 98 °C for 30 s; 32 cycles of 98 °C for 10 s, 54 °C for 30 s, and 72 °C for 45 s; and a final extension at 72 °C for 10 min. PCR products were purified using AMPure XT magnetic beads (Beckman Coulter Genomics, Danvers, MA, USA), quantified using Qubit, evaluated with an Agilent 2100 Bioanalyzer (Agilent, USA) and an Illumina library quantification kit (Kapa Biosciences, Woburn, MA, USA), pooled, and sequenced on an Illumina NovaSeq 6000 platform (PE250).

Demultiplexed raw reads were trimmed to remove primer sequences using cutadapt (v1.9). Paired-end reads were merged with FLASH (v1.2.8). Low-quality reads (quality score < 20), short reads (< 100 bp), and reads containing > 5% ambiguous bases (“N”) were filtered using the sliding window algorithm in fqtrim (v0.94), yielding high-quality clean tags. Chimeric sequences were identified and removed using Vsearch (v2.3.4). Denoising and amplicon sequence variant (ASV) inference were performed with DADA2. Taxonomic annotation was carried out using the QIIME2 feature-classifier plugin against the SILVA database (v138). Alpha and beta diversity indices were calculated in QIIME2, and relative abundances were used for taxonomic profiling. Differentially abundant genera were identified using the Wilcoxon test (p < 0.05). Linear discriminant analysis effect size (LEfSe; LDA ≥ 3.0, p < 0.05) was performed using the nsegata-lefse pipeline. Additional plots were generated using R (v3.4.4).

### 2.15 Statistical analysis

Data are presented as mean ± standard deviation (SD). The Shapiro–Wilk test was used to assess normality, and Levene’s test was used to evaluate homogeneity of variance. Differences among multiple groups were analyzed using one-way analysis of variance (ANOVA) followed by Tukey’s *post hoc* test. A p value < 0.05 was considered statistically significant. All graphs were generated using GraphPad Prism software (v10.1.2).

Survival curves were compared using the stratified log-rank test (stratified by experiment batch), and hazard ratios (HR) with 95% CI were estimated by Cox regression (28). Multiple comparisons across extensive endpoints were controlled using the Benjamini–Hochberg false discovery rate (FDR) at q < 0.05. Sensitivity analyses (ITT-like censoring and worst-case imputation) including all mice were performed to assess the impact of exclusion criteria (29).

## 3 Results

### 3.1 CEH improves survival and alleviates clinical severity in Salmonella-infected adult mice

Of the total mice enrolled, 4 (6.3%) met the predefined exclusion criteria and were excluded from the primary analysis but included in sensitivity analyses (**Supplementary Figure 1**). No significant differences in baseline body weight or clinical scores were observed between excluded and included mice (**Supplementary Table 1**).

**Figure 1:**
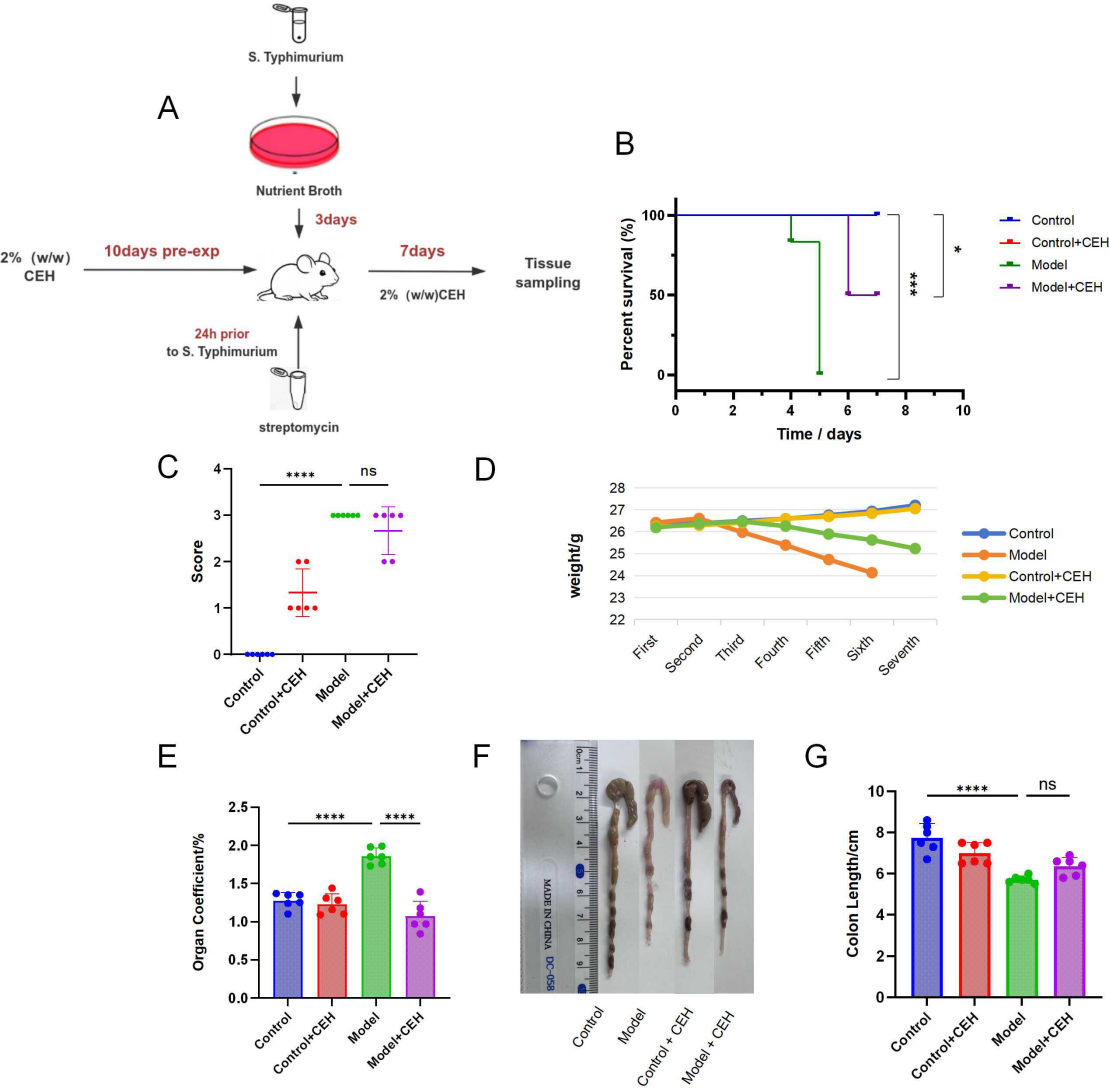
Effects of CEH on body condition and visceral function in adult mice. **(A)** Experimental Flowchart; **(B)** Survival curves in adult mice(n = 6); **(C)** DAI scores in adult mice(n = 6); **(D)** Body weight changes in adult mice(n = 6); **(E)** Spleen weight/body weight ratio in adult mice(n = 6); **(F, G)** Colon length in adult mice; Data are presented as mean ± SD. Significance was accepted at *p < 0.05, **p < 0.01, ***p < 0.001,****p < 0.0001.

As shown in **Figure 1A**, a S. Typhimurium–induced colitis model was first established in adult mice, and the protective effect of CEH was evaluated on this basis. Oral CEH markedly attenuated the clinical progression of Salmonella-induced colitis. Survival analysis revealed a sharp decline in survival in the model group after infection, whereas survival was notably improved in the model + CEH group (log-rank test; HR = 0.17, 95% CI:0.04–0.71, p = 0.015). (**Figure 1B**). Consistently, the disease activity index (DAI) was significantly increased in the model group compared with the control group (p < 0.0001), reflecting aggravated diarrhea and bloody stools, while DAI in the model + CEH group showed only a mild, non-significant reduction relative to the model group (**Figure 1C**). After infection, body weight continuously decreased in the model group, whereas CEH treatment markedly alleviated weight loss; CEH alone (control + CEH vs. control) did not cause obvious adverse changes in body weight (**Figure 1D**).

Organ index analysis further supported this protective effect. Compared with the control group, spleen index was significantly elevated in the model group (p < 0.0001), indicating an increased systemic inflammatory burden; CEH intervention markedly reduced spleen index after infection (p < 0.0001) (**Figure 1E**). Colonic length was significantly shortened in adult mice of the model group (p < 0.0001), whereas colonic length in the model + CEH group showed a trend toward recovery, although the difference did not reach statistical significance (**Figures 1F, G**).

### 3.2 Casein hydrolysate preserves intestinal mucosal architecture and goblet cell integrity

**Figure 2:**
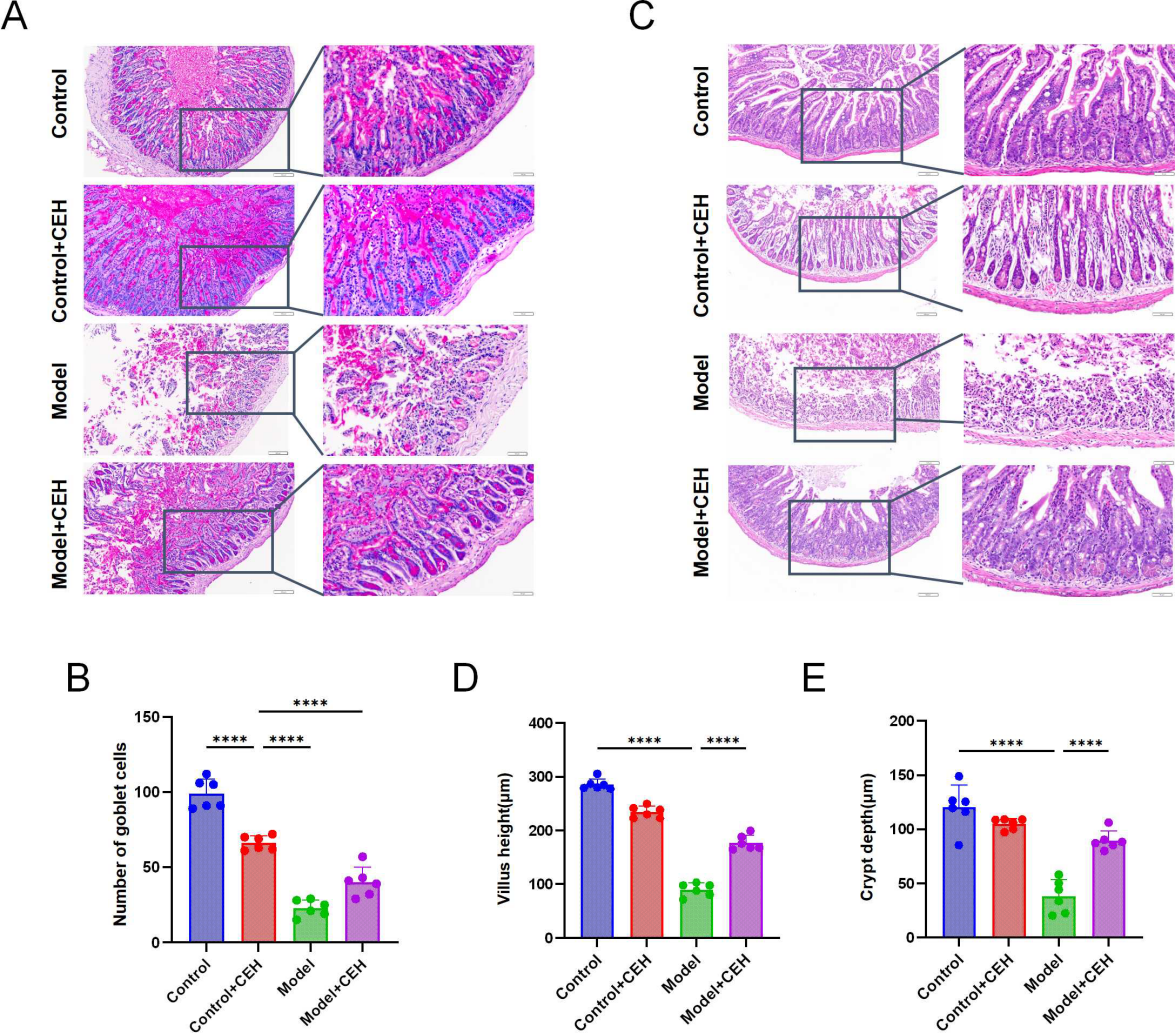
CEH preserves ileal mucosal architecture and goblet cell integrity in adult mice. with Salmonella-induced colitis **(A)** PAS staining of ileum (20× magnification, scale bar = 50 μm); **(B)** Goblet cell count in adult mice(n = 6). **(C)** H&E staining of ileum (20×, scale bar = 50 μm); **(D)** Villus length in adult mice(n = 6). **(E)** Crypt depth in adult mice(n = 6).

At the histological level, PAS staining of the ileum showed that the number of goblet cells in the crypt region was markedly lower in the model group than in the control group, indicating damage to the mucus-secreting barrier (**Figure 2A**). In contrast, the number of goblet cells in the model + CEH group was significantly higher than in the model group (p < 0.01), and the difference from the control group was narrowed (**Figure 2B**).

H&E staining further verified the protective effect of CEH on mucosal structure. In the model group, ileal villi were obviously atrophic, crypts became shallow, epithelial architecture was disorganized, and massive inflammatory cell infiltration was observed (**Figure 2C**). Quantitative analysis showed that villus length was significantly shortened (p < 0.0001) and crypt depth was markedly decreased (p < 0.0001) in the model group compared with controls (**Figures 2D, E**). After CEH treatment, both indices were significantly improved, with villus length and crypt depth (both p < 0.0001 vs. model) markedly increased and approaching control levels. Collectively, these results indicate that CEH preserves villus–crypt architecture and goblet cell function in adult mice during Salmonella-induced colitis.

### 3.3 CEH improves the intestinal inflammatory milieu and reduces mucosal immune cell infiltration

**Figure 3:**
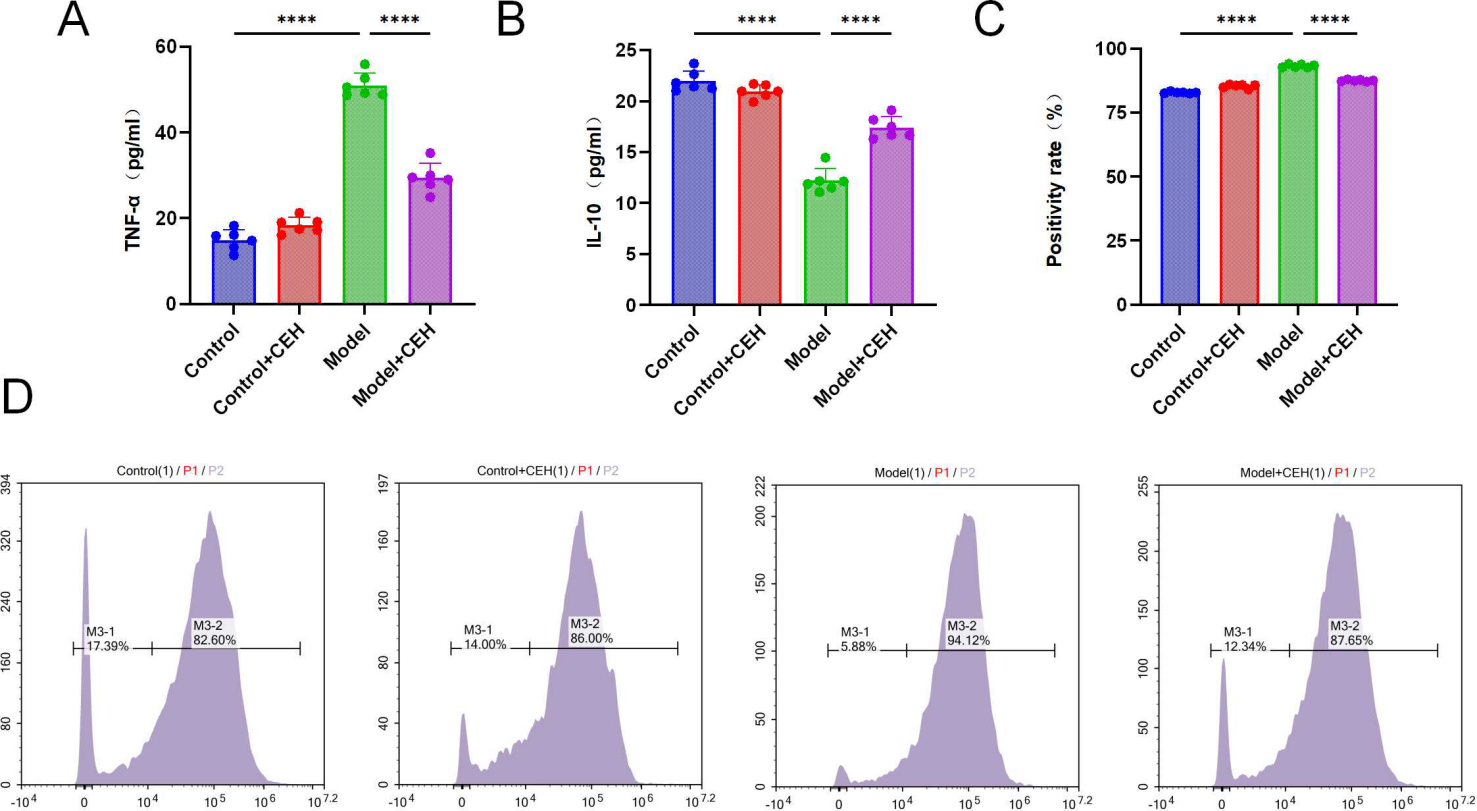
CEH attenuates intestinal inflammation and CD45^+^ immune cell infiltration in adult mice. **(A)** TNF-α level in adult mice (n = 6); **(B)** IL-10 level in adult mice (n = 6); **(C)** CD45^+^ flow plots; **(D)** CD45^+^ cell proportion (n = 6);Data are mean ± SD. Significance was accepted at *p < 0.05, **p < 0.01, ***p < 0.001,****p < 0.0001.

ELISA results showed that levels of the pro-inflammatory cytokine Tumor Necrosis Factor-α(TNF-α) in ileal tissue of the model group were significantly elevated compared with controls (p < 0.0001) (**Figure 3A**), whereas levels of the anti-inflammatory cytokine Interleukin-10(IL-10) were significantly reduced (p < 0.0001) (**Figure 3B**). In the model + CEH group, TNF-α levels were significantly lower than in the model group (p < 0.0001), While IL-10 levels were significantly increased relative to the model group (p < 0.0001), suggesting that CEH modulates local cytokine homeostasis to exert anti-inflammatory effects.

To evaluate immune cell infiltration in the intestinal mucosa, the proportion of CD45^+^(cluster of differentiation 45, pan-leukocyte marker) cells in intestinal tissue was analyzed by flow cytometry (**Figures 3C, D**). Compared with the control group, the model group displayed a significant accumulation of CD45^+^ immune cells. Following CEH intervention, the proportion of CD45^+^ cells in the model + CEH group was markedly reduced, approaching the level of uninfected controls (p < 0.0001) (**Figure 3D**). These findings suggest that CEH effectively inhibits excessive recruitment of inflammatory cells into the intestinal mucosa, in line with the down-regulation of inflammatory cytokines detected by ELISA.

### 3.4 CEH promotes intestinal stem cell regeneration

**Figure 4:**
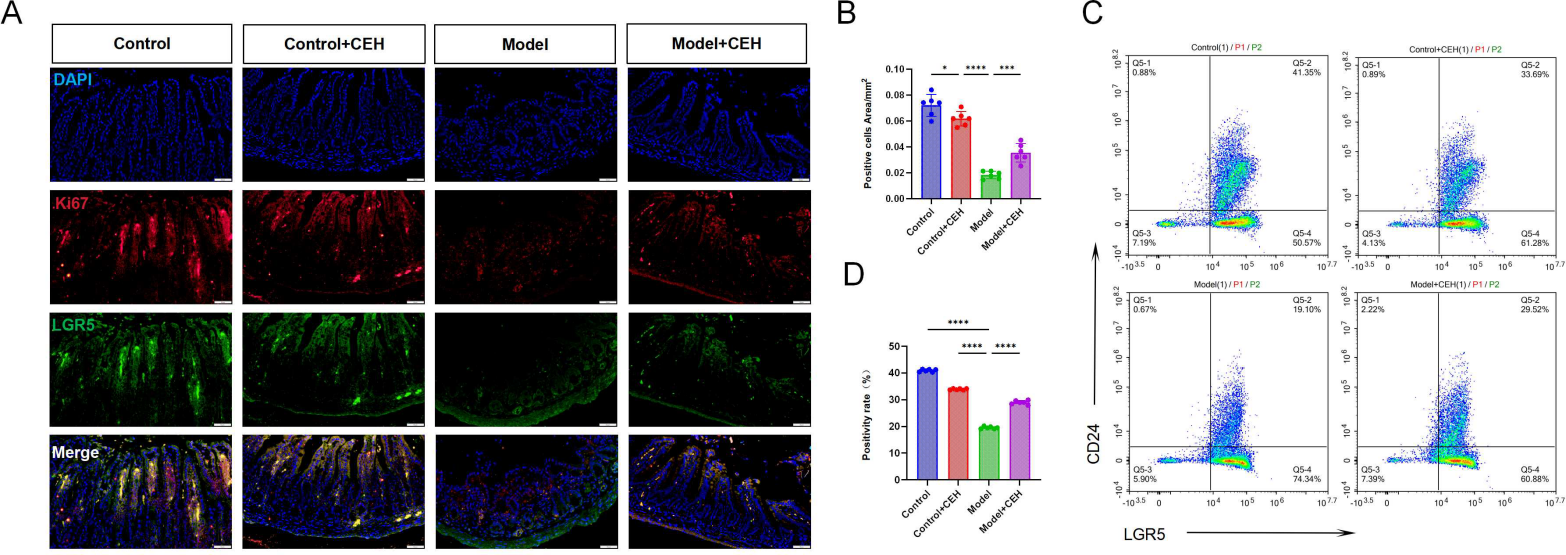
CEH promotes intestinal LGR5^+^ stem cell regeneration and reconstruction of highly proliferative subpopulations in adult mice. **(A)** LGR5^+^Ki67^+^ immunofluorescence (20×, scale bar = 50 μm); **(B)** Positive cells areas for LGR5^+^Ki67^+^(n = 6); **(C)** Flow plots of CD24^+^LGR5^+^ cells; **(D)** Positive rate of CD24^+^LGR5^+^(n = 6). Data are mean ± SD. Significance was accepted at *p < 0.05, **p < 0.01, ***p < 0.001,****p < 0.0001.

To assess the impact of CEH on epithelial regeneration, double immunofluorescence staining for LGR5 and Ki67 was performed in ileal sections from adult mice (**Figure 4A**). After Salmonella infection, the number of LGR5^+^ stem cells in ileal crypts of the model group was markedly reduced, and their co-localization with Ki67 was significantly decreased (p < 0.0001) (**Figure 4B**). In contrast, the model + CEH group showed a notable increase in LGR5^+^/Ki67^+^ double-positive cells in the crypt region (p < 0.001 vs. model), indicating that CEH exerts an “activation of regeneration” effect under infectious stress.

Subsequent flow cytometry analysis further confirmed these trends at the cellular level (**Figure 4C**). Dual staining for CD24 and LGR5 showed that the proportion of the CD24^+^LGR5^+^ stem cell subpopulation in the intestine was significantly reduced in the model group (p < 0.0001), whereas this proportion was markedly increased in the model + CEH group (p < 0.0001) (**Figure 4D**). These data indicate that CEH not only increases the overall number of LGR5^+^ intestinal stem cells but also facilitates the reconstruction of a highly proliferative CD24^+^LGR5^+^ stem cell compartment.

### 3.5 CEH enhances intestinal barrier integrity and nutrient transport function

**Figure 5:**
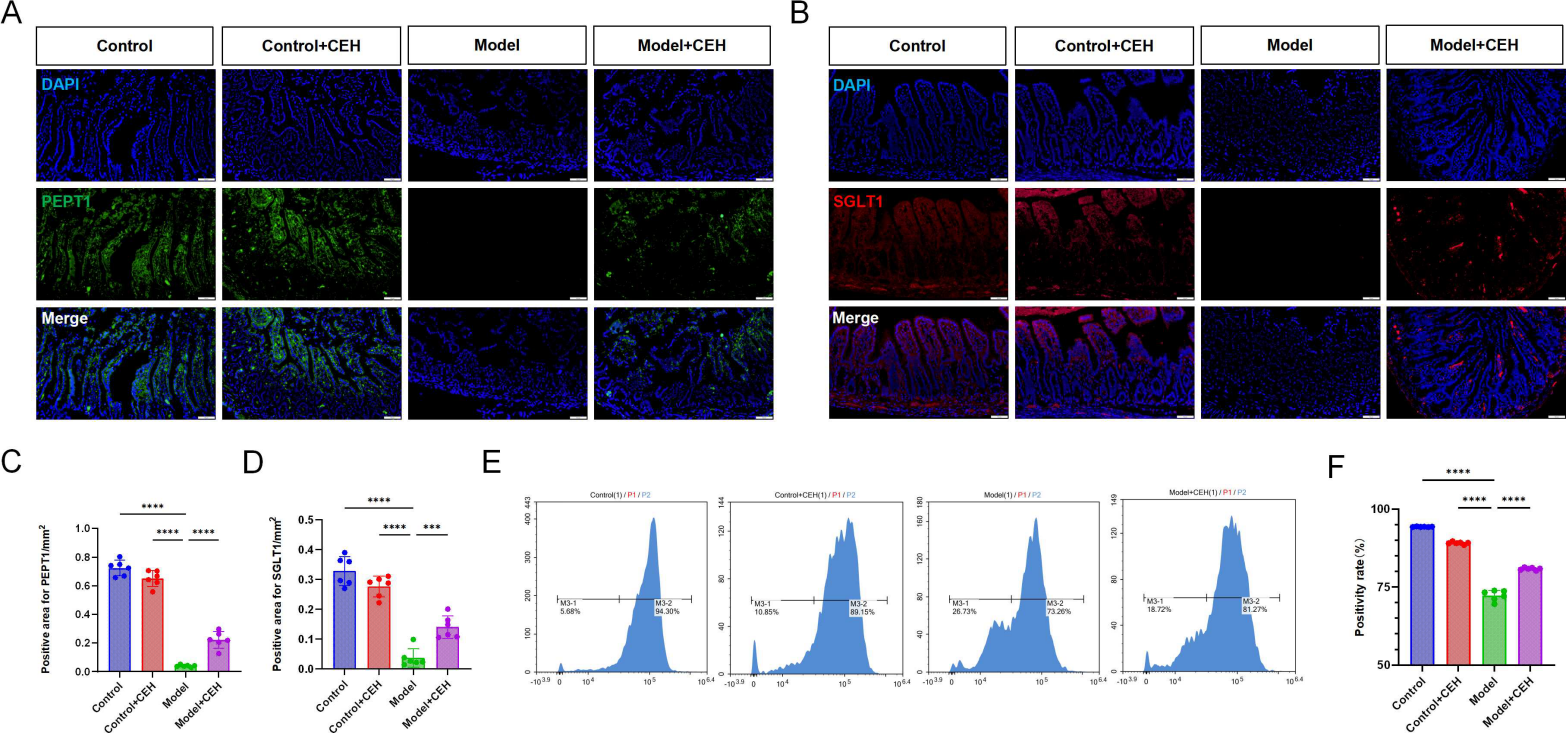
CEH restores the expression of ileal nutrient transporters and adherens junction proteins in adult mice. **(A)** PEPT1 immunofluorescence (20×, scale bar = 50 μm); **(B)** SGLT1 immunofluorescence (20×, scale bar = 50 μm); **(C)** Positive cells areas for PEPT1 (n = 6); **(D)** Positive cells areas for SGLT1 (n = 6); **(E)** Flow plots of E-cadherin^+^ cells (n = 6); **(F)** Positive rate of E-cadherin (n = 6). Data are mean ± SD. Significance was accepted at *p < 0.05, **p < 0.01, ***p < 0.001,****p < 0.0001(n=6).

Immunofluorescence staining showed that the numbers of PEPT1^+^(peptide transporter 1), and SGLT1^+^ (sodium-glucose co-transporter 1) epithelial cells in the ileum of adult mice were significantly decreased in the model group (**Figures 5A–D**, both p < 0.0001 vs. control). In the model + CEH group, the numbers of PEPT1^+^ (p < 0.0001) and SGLT1^+^ (p < 0.001) cells were significantly increased compared with the model group, and positive signals again displayed a continuous distribution along villus and crypt epithelium (**Figures 5A–D**). These findings indicate that CEH primarily exerts a “restoration of nutrient transport function” under infectious stress.

In addition, the proportion of E-cadherin^+^(epithelial cadherin) epithelial cells in intestinal tissue was assessed by flow cytometry (**Figure 5E**). The model group showed a marked reduction in the proportion of E-cadherin^+^ cells compared with controls (p < 0.0001), reflecting disruption of intercellular junctions. Following CEH intervention, the proportion of E-cadherin^+^ cells in the model + CEH group was significantly increased and clearly higher than in the model group (p < 0.0001) (**Figure 5F**). Together, these results suggest that CEH effectively reverses infection-induced loss of junctional proteins and preserves epithelial barrier integrity.

### 3.6 CEH reshapes gut microbiota and significantly enriches lactic acid–producing bacteria

**Figure 6:**
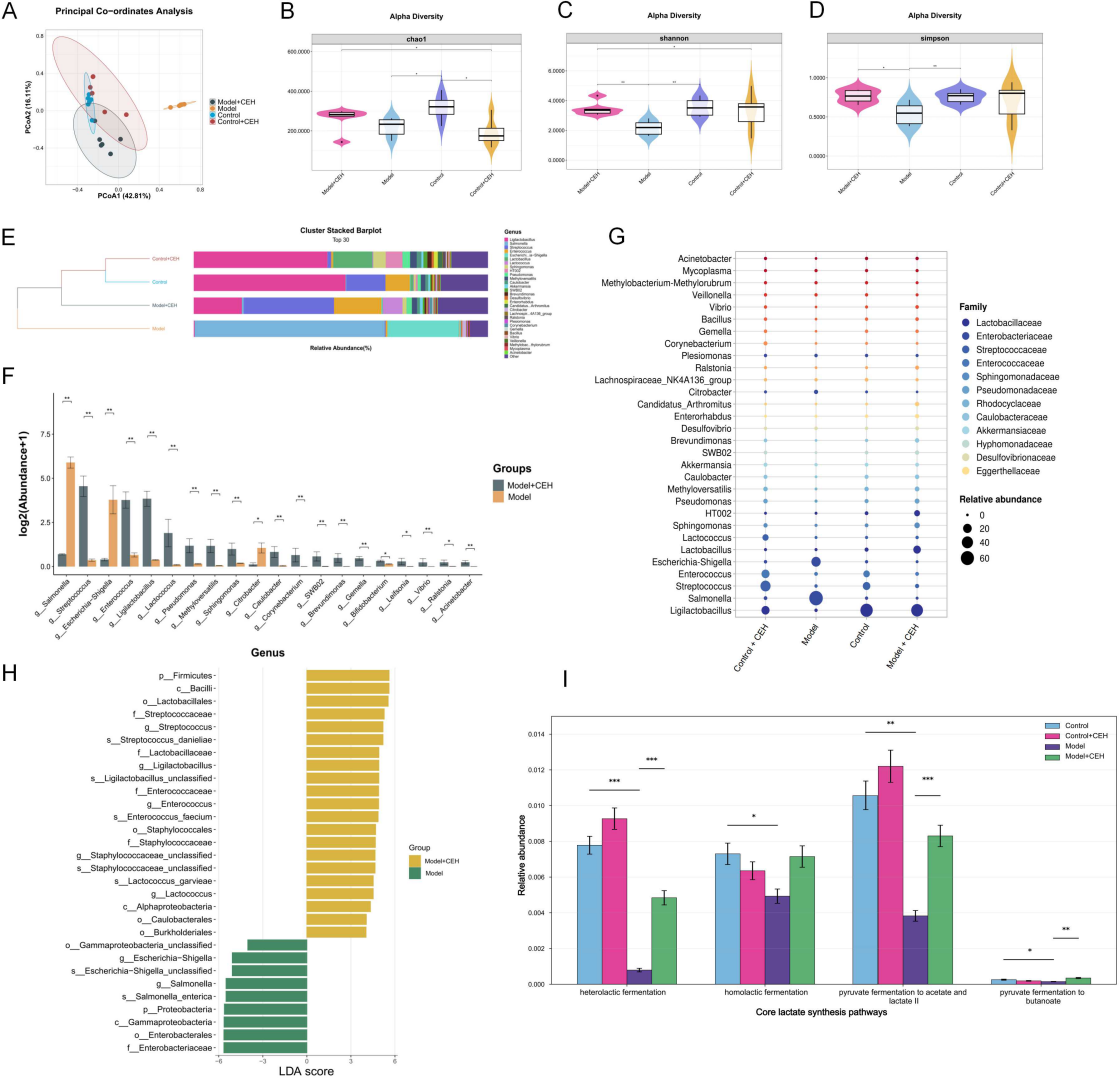
CEH reshapes gut microbiota and enriches lactate-producing genera in adult colitis mice. **(A)** PCoA analysis based on Bray-Curtis distance (n = 6); **(B–D)** Gut microbial alpha diversity (Chao1, Shannon, Simpson indices, n=6); **(E)** Genus-level sample cluster stacked barplot based on Bray-Curtis distance (n = 6); **(F)** Genus-level differential analysis between model+CEH and model groups (n = 6); **(G)** Family-level bubble plot (n = 6); **(H)** LDA score plot of model+CEH vs. Model (n = 6). **(I)** Effects of CEH intervention on the relative abundance of core lactate synthesis pathways in gut microbiota (n = 6). Significance was accepted at *p < 0.05, **p < 0.01, ***p < 0.001,****p < 0.0001.

To evaluate the impact of CEH on intestinal microecology in Salmonella-induced colitis, 16S rDNA sequencing was performed on intestinal contents from each group. Principal coordinates analysis (PCoA) showed that samples from the model group were clearly separated from those of the control group in two-dimensional space, indicating pronounced infection-induced dysbiosis (**Figure 6A**). By contrast, the sample points of the model + CEH group clustered closer to the control group and were separated from the model group, suggesting that CEH intervention can partially reverse infection-induced microbiota disruption.

Alpha diversity indices, including chao1, shannon, and simpson, were calculated to assess gut microbial richness and diversity across groups. Compared with the control group, the model group exhibited a significant reduction in both microbial richness and diversity (Chao1, p<0.05; shannon, p<0.01; and simpson, p<0.01). Notably, the model+CEH group showed a significant recovery in the shannon (p<0.01 vs. model) and simpson (p<0.05 vs. model) indices, alongside improving trends in the chao1 index, indicating that CEH intervention effectively restored the reduced gut microbial diversity in the model mice(**Figures 6B–D**).

Cluster analysis yielded similar results: samples from the model group formed a distinct branch, clearly separated from the control and control + CEH groups, whereas the model + CEH group clustered nearer to the controls (**Figure 6E**).

Genus-level compositional analysis showed that, compared with the model group, CEH intervention significantly reduced the abundance of pathogenic genera (g_Salmonella, p < 0.01; g_Escherichia-Shigella, p < 0.01), while significantly increasing g_Ligilactobacillus (p < 0.01), g_Streptococcus (p < 0.01), g_Enterococcus (p < 0.01), g_Lactococcus (p < 0.01), and other lactic acid–producing taxa (**Figure 6F**).

At the family level, the relative abundance of harmful or opportunistic bacteria, such as Salmonella, was increased in the model group. After CEH treatment, the relative abundances of Ligilactobacillus, Streptococcus, Enterococcus, Lactococcus and other genera were significantly increased in the model + CEH group and were much higher than in the model group (**Figure 6G**). Further LEfSe-LDA analysis showed that these genera with the most pronounced abundance changes also had high LDA scores (LDA > 3) (**Figure 6H**), identifying them as key discriminative taxa among groups.

Functional prediction of core lactate synthesis pathways revealed that the activities of key pathways, including heterolactic fermentation (p<0.001) and pyruvate fermentation to acetate and lactate II (p<0.01), were significantly downregulated in the model group relative to the control group. In contrast, these pathways were significantly upregulated in the model+CEH group (both p<0.001 vs. model). These findings suggest that CEH modulates the lactate metabolism function of the gut microbiota, thereby restoring intestinal microenvironmental homeostasis.

### 3.7 CEH increases lactate levels and is associated with improved Paneth cell–Wnt niche function

**Figure 7:**
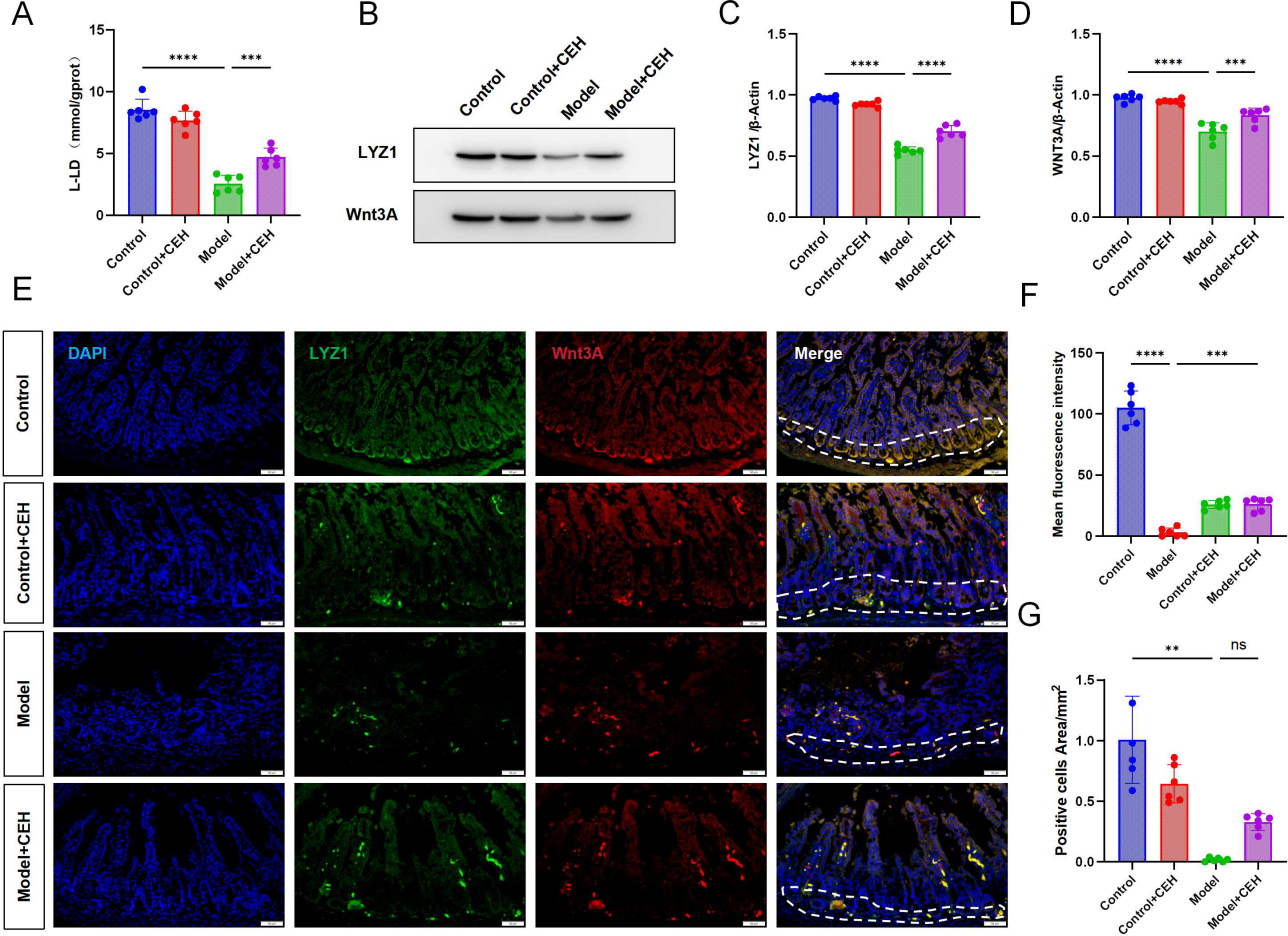
CEH is associated with enhanced intestinal mucosal repair and activation of the lactate–Paneth cell–Wnt axis in adult mice. **(A)** Levels of lactate in the intestinal tract (n = 6); **(B)** Western blot analysis of LYZ1 and Wnt3A; **(C, D)** LYZ1 and Wnt3A protein quantification (n = 6). Data are mean ± SD. Significance was accepted at *p < 0.05, **p < 0.01, ***p < 0.001,****p < 0.0001(n=3). **(E)** LYZ1^+^Wnt3A^+^ immunofluorescence (20×, scale bar = 50 μm); **(F)** Mean fluorescence intensity (MFI) of LYZ1^+^Wnt3A^+^ (n = 6); **(G)** Positive cells areas for LYZ1^+^Wnt3A^+^ (n = 6). Significance was accepted at *p < 0.05, **p < 0.01, ***p < 0.001,****p < 0.0001.

To link microbiota changes with metabolic output, L-lactate concentrations in intestinal contents were measured. L-lactate levels in the model + CEH group were significantly higher than those in the model group (p < 0.0001) (**Figure 7A**), consistent with the aforementioned enrichment of lactate bacteria. This observation suggests that CEH-related microbiota remodeling is accompanied by enhanced lactate production, which may contribute to a microenvironment favorable for epithelial repair.

At the protein level, compared with the control group, expression of LYZ1 and Wnt3A in the ileum of adult mice in the model group was significantly down-regulated (both p < 0.0001), whereas their expression in the model + CEH group was significantly increased (both p < 0.0001) (**Figures 7B–D**).

Immunofluorescence staining showed that CEH was associated with restored LYZ1 and Wnt3A signals in the crypt niche and expanded LYZ1^+^/Wnt3A^+^ double-positive areas(**Figure 7E**), In the model group, LYZ1 fluorescence intensity was clearly reduced compared with the control group, with a marked decrease in the number of LYZ1^+^ cells; Wnt3A signal was likewise markedly diminished, and the area of co-localization between the two markers was reduced (both p < 0.0001 vs. control) (**Figures 7F, G**); Compared with the model group, the model + CEH group exhibited significantly increased fluorescence intensities of LYZ1 and Wnt3A in the crypt region, along with a marked expansion of their co-localized area; the proportion of LYZ1^+^/Wnt3A^+^ cells was significantly higher than in the model group (p < 0.0001) (**Figures 7F, G**).

These observations indicate that CEH is accompanied by improved Paneth cell function and enhanced Wnt-related support for intestinal stem cells.

### 3.8 The effects of CEH on intestinal homeostasis in Salmonella-infected mice via lactate-GPR81/Wnt axis

**Figure 8:**
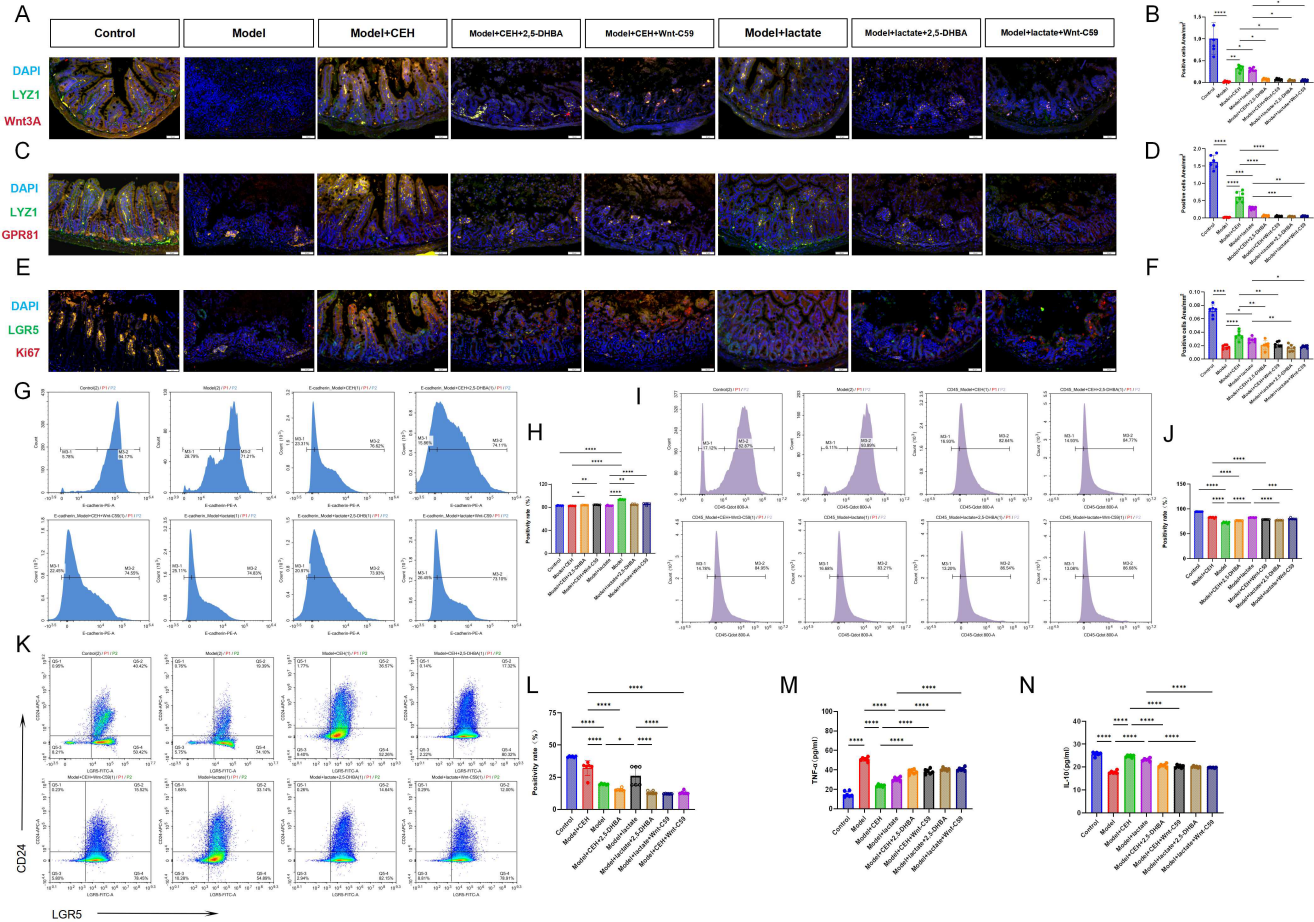
CEH improves intestinal epithelial regeneration, barrier function, and immune homeostasis in Salmonella-infected adult mice. **(A, B)** Analysis of LYZ1^+^Wnt3A^+^ immunofluorescence staining outcomes (20×, scale bar = 50 μm, n=6); **(C, D)** Analysis of LYZ1^+^GPR81^+^ immunofluorescence staining outcomes (20×, scale bar = 50 μm, n=6); **(E, F)** Analysis of LGR5^+^Ki67^+^ immunofluorescence staining outcomes (20×, scale bar = 50 μm, n=6); **(G, H)** Analysis of E-cadherin^+^ flow cytometry staining results (n=6); **(I, J)** Analysis of CD45^+^ flow cytometry staining results (n=6); **(K, L)** Analysis of CD24^+^LGR5^+^ flow cytometry staining results (n=6); **(M)** TNF-α level in adult mice (n = 6); **(N)** IL-10 level in adult mice (n = 6). Significance was accepted at *p < 0.05, **p < 0.01, ***p < 0.001,****p < 0.0001.

To explore the potential underlying mechanism by which CEH alleviates intestinal damage, exogenous lactate supplementation and pharmacological blockade experiments were performed (**Figure 8**). Immunofluorescence results showed that compared with the model group, both CEH and exogenous lactate supplementation significantly elevated these protein levels to improve paneth cell function and stem cell proliferation: LYZ1^+^/Wnt3A^+^(**Figures 8A, B**; model vs. model + CEH, p < 0.01; model vs.model+lactate, p < 0.05), LYZ1^+^/GPR81^+^ (**Figures 8C, D**; model vs. model + CEH, p < 0.0001; model vs.model+lactate, p < 0.001)and Lgr5^+^/Ki67^+^(**Figures 8E, F**; model vs. model + CEH, p < 0.0001; model vs.model+lactate, p < 0.05). Notably, compared with the single CEH or lactate treatment groups, the combined intervention with 2,5-DHBA or Wnt-C59 obviously reversed the above improvements, indicating that blockage of GPR81 or Wnt signaling eliminated the protective effects of CEH and lactate.

Flow cytometry analysis further validated intergroup differences. The CEH and lactate treatment exhibited higher E-cadherin^+^ (**Figures 8G, H**; model vs. model + CEH, p<0.0001; model vs.model+lactate, p < 0.0001) epithelial cells and LGR5^+^CD24^+^ (**Figures 8K, L**; model vs. model + CEH, p < 0.0001; model vs. model+lactate, p < 0.01) stem cells, along with reduced CD45^+^ (**Figures 8I, J**; model vs.model + CEH, p < 0.0001; model vs.model+lactate, p < 0.0001) inflammatory cells relative to the model group. Such amelioration was reduce in inhibitor-treated groups. Consistent with the above results, ELISA assays revealed that the model+CEH and model+lactate group had lower TNF-α (**Figure 8M**) and higher IL-10 (**Figure 8N**) than the model group. whereas inhibitor intervention deteriorated inflammation again compared with the CEH and lactate monotherapy groups.

## 4 Discussion

This study demonstrates that dietary supplementation with CEH confers robust protection against S. Typhimurium–induced enteritis (30) in adult mice. CEH alleviates clinical disease, attenuates systemic and local inflammation, promotes intestinal stem cell-driven epithelial regeneration, and preserves mucosal barrier integrity. Mechanistically, we establish a causal axis whereby CEH-enriched lactate-producing bacteria elevate luminal lactate, which activates Paneth cell GPR81 signaling to upregulate Wnt3A, thereby driving ISC-mediated epithelial repair.

At the clinical level, CEH markedly improved survival in adult mice challenged with S. Typhimurium, reduced DAI scores, and alleviated infection−induced weight loss, while CEH alone did not induce adverse changes in body weight. These findings indicate that CEH is both efficacious and well tolerated in adult hosts. Organ index analysis revealed pronounced splenomegaly in infected mice, reflecting a heightened systemic inflammatory burden; CEH significantly reduced the spleen index, suggesting mitigation of systemic inflammatory stress (31, 32). Infection also caused substantial shortening of the colon, whereas CEH tended to normalize colonic length, indicating a global protective effect on the inflamed intestine. Histologically, infected adult mice displayed severe ileal villus atrophy, shallow crypts, disorganized epithelial architecture, and massive inflammatory cell infiltration (30, 33–35). CEH treatment preserved villus morphology, improved crypt structure, and reduced inflammatory infiltration. PAS staining further showed a marked loss of goblet cells in the crypt region of infected mice, indicating damage to the mucus−secreting barrier, whereas CEH significantly restored goblet cell numbers and partially normalized the mucus layer. Collectively, these results demonstrate that CEH not only improves clinical outcomes but also substantially attenuates structural damage to the adult intestinal mucosa during Salmonella−induced colitis.

At the level of the local intestinal environment, CEH exerted a pronounced anti−inflammatory effect. In ileal tissue from infected adult mice, TNF−α levels were significantly elevated and IL−10 levels were markedly reduced, reflecting a highly pro−inflammatory milieu. CEH treatment significantly lowered TNF−α and restored IL−10, shifting the cytokine profile toward an anti−inflammatory and tissue−reparative state (36, 37). In parallel, flow cytometric analysis revealed substantial accumulation of CD45^+^ leukocytes in the intestinal mucosa of infected mice, whereas CEH significantly reduced the proportion of CD45^+^ cells, nearly to control levels. These data indicate that CEH dampens excessive immune cell recruitment and resolves inflammatory responses in the adult intestine following bacterial challenge. CEH also promoted functional restoration of the epithelial barrier. Infection led to a marked reduction in PEPT1^+^ and SGLT1^+^ epithelial cells, consistent with impaired nutrient transport capacity. In the CEH−treated infected group, the numbers of PEPT1^+^ and SGLT1^+^ cells were significantly increased, and their expression again showed continuous distribution along villi and crypts, indicating recovery of absorptive and transport functions (38, 39). Furthermore, the proportion of E−cadherin^+^ epithelial cells was significantly reduced in infected mice, suggesting disruption of adherens junctions and barrier integrity (40); CEH treatment restored E−cadherin expression and substantially reversed this barrier disruption. Thus, CEH enhances barrier function in adult Salmonella colitis by concurrently limiting inflammatory injury and promoting the structural and functional integrity of the epithelium.

A key mechanistic dimension of this work is the impact of CEH on intestinal microbiota composition. 16S rDNA sequencing revealed that Salmonella infection induced a profound dysbiosis in adult mice, with clear separation between control and model groups in PCoA plots and distinct clustering at the community level (41). CEH treatment partially reversed this dysbiosis: the microbiota profile of the model + CEH group shifted toward that of uninfected controls in both ordination and clustering analyses,consistent with previous reports that casein hydrolysates can normalize disturbed gut microbial communities (17). At the compositional level, infection increased the relative abundances of pathogenic or opportunistic taxa such as Salmonella and Escherichia–Shigella (41). CEH markedly re duced these pathogenic genera while selectively enriching multiple lactic acid–producing genera, including Ligilactobacillus, Streptococcus, Enterococcus, and Lactococcus, in agreement with findings that casein hydrolysates selectively support LAB growth and suppress enterobacteria (24). LEfSe analysis confirmed that these lactic acid–producing taxa were among the key discriminant features in CEH−treated animals. Importantly, the enrichment of lactic acid bacteria was functionally reflected by significantly increased L−lactate concentrations in intestinal contents of the model + CEH group compared with the infection−only group. These findings indicate that CEH not only shifts microbial composition but also enhances the metabolic activity of lactic acid bacteria, establishing a lactate−rich luminal environment that is conducive to epithelial repair and energy supply (42, 43).

We next investigated whether this elevated lactate directly contributes to epithelial repair. Exogenous L−lactate supplementation in infected mice phenocopied CEH’s effects: it increased LYZ1^+^/Wnt3A^+^ co−localization, expanded LGR5^+^/Ki67^+^ proliferating ISCs, restored E−cadherin^+^ epithelial cells, reduced CD45^+^ leukocyte infiltration, and normalized TNF−α/IL−10 balance. These data demonstrate that lactate alone is sufficient to drive the regenerative and anti−inflammatory phenotypes seen with CEH. To identify the molecular sensor of lactate, we examined GPR81, a well-known lactate sensing receptor (44). Double immunofluorescence revealed that LYZ1^+^ Paneth cells co-express GPR81 (**Figures 8C, D**), providing the first evidence that Paneth cells can directly sense lactate. Pharmacological blockade of GPR81 using 2,5-DHBA significantly reversed the protective effects of both CEH and exogenous lactate, abolishing the improvements in Paneth cell function, ISC proliferation, barrier integrity, and inflammatory cytokines (**Figure 8**). Similarly, inhibition of Wnt secretion with Wnt-C59 eliminated the regenerative benefits of CEH and lactate, placing Wnt3A downstream of GPR81 activation.

The consequences of Paneth cell–Wnt axis restoration were evident at the level of ISCs (9). Salmonella infection markedly reduced the number of LGR5^+^ stem cells and LGR5^+^/Ki67^+^ proliferating cells in ileal crypts of adult mice, indicating a severe compromise of epithelial self−renewal capacity. CEH treatment significantly increased the number of LGR5^+^/Ki67^+^ double−positive cells, reflecting reactivation of ISC proliferation under infectious stress. Flow cytometric analysis further showed that the proportion of CD24^+^LGR5^+^ cells-a highly proliferative ISC subpopulation (45), was significantly depleted by infection but robustly restored and expanded by CEH. Notably, lactate supplementation alone reproduced this ISC expansion, while GPR81 or Wnt blockade abrogated it (**Figures 8E, F, K, L**), confirming that lactate acts via GPR81 and Wnt to drive ISC regeneration. The subsequent upregulation of nutrient transporters (PEPT1, SGLT1) and the adhesion molecule E−cadherin further indicates that CEH not only boosts ISC proliferation but also supports the differentiation and functional maturation of regenerated epithelium, leading to durable restoration of barrier function in adult hosts (38, 39).

Our findings indicate that effective management of the adult gut requires not only pathogen clearance but also active support of microbiota homeostasis, metabolic signaling, and stem cell niche function. The benefits of CEH, particularly through a microbiota-derived lactate-GPR81-Wnt3A-ISC axis, highlight the potential of targeted nutritional interventions to correct age-related defects in intestinal repair (19, 46, 47). By concurrently reshaping the microbiota, boosting beneficial metabolites, and reactivating regenerative signaling pathways, CEH acts on multiple levels of age-associated intestinal fragility.

This study has several limitations. First, CEH is a complex mixture, and the specific bioactive peptide components responsible for microbiota modulation, Paneth cell activation, and ISC stimulation remain to be identified. Peptidomic profiling and functional validation of individual peptides will be required to pinpoint the key active sequences. Second, the use of streptomycin pretreatment to facilitate Salmonella colonization, while a standard model, substantially disrupts the gut microbiota and creates an artificial ecological state. Therefore, the observed microbiota alterations associated with CEH treatment may partly reflect post-antibiotic recolonization dynamics rather than selective modulation by CEH. Future studies using germ-free mice, co-housing, or non-antibiotic infection models are needed to establish causality. Third, although we demonstrated Gpr81 expression on Paneth cells and used a specific inhibitor, we did not perform genetic Gpr81 knockout experiments; thus, off-target effects of 2,5-DHBA cannot be completely excluded. Future studies using Paneth cell-specific Gpr81 knockout mice would provide definitive proof. Finally, translation of these findings to humans will require careful evaluation of dosing, safety, and efficacy in clinical populations.

## 5 Conclusions

In conclusion, the present study demonstrated that CEH exerted prominent protective effects against S. Typhimurium-induced intestinal injury in mice. CEH reshaped gut microbiota composition and increased the abundance of lactate-producing bacteria, which contributed to the elevation of intestinal lactate content. The increased lactate level was closely correlated with improved Paneth cell function, activated intestinal stem cell proliferation, and maintained epithelial barrier integrity, accompanied by the upregulation of GPR81 and Wnt3A. Consistent with these observations, exogenous lactate supplementation and pharmacological intervention assays indicated that lactate and Wnt signaling were closely associated with the intestinal protective capacity of CEH. Collectively, CEH alleviated intestinal inflammation and effectively restored intestinal homeostasis. This work provides novel insights into the protective function of CEH and supports CEH as a promising nutritional strategy for protecting the intestine against bacterial infection.

## Data Availability

The datasets presented in this study can be found in online repositories. The names of the repository/repositories and accession number(s) can be found below: https://www.ncbi.nlm.nih.gov/, PRJNA1420017.
