# Supplementary material for: Casein hydrolysate promotes intestinal repair in adult mice with Salmonella enteritis through a lactate-GPR81-Wnt3A axis in intestinal stem cell niche
Source: Front Immunol. 2026 Jun 15;17:1817158. doi: 10.3389/fimmu.2026.1817158 (PMC13310695; doi:10.3389/fimmu.2026.1817158)
Supplement: Supplementary file 1 [file SupplementaryFile1.docx]

Figure S1.ITT-like sensitivity analysis of survival in mice with Salmonella infection.Significance was accepted at *p < 0.05, **p < 0.01, ***p < 0.001,****p < 0.0001

Table S1.Characteristics of mice excluded from the primary analysis.

| Mouse ID | Group | Exclusion criterion | Fecal Salmonella load at 48h (CFU/g) | Body weight loss at exclusion (%) | DAI score at exclusion | Day of exclusion |
| --- | --- | --- | --- | --- | --- | --- |
| M3 | Model | Persistently low load | 1.8×10^3^ | -0.3% | 1 | 4 |
| M6 | Model | No detectable Salmonella | ＜10^2^ | 3.5% | 1 | 2 |
| MC1 | Model+CEH | Persistently low load | 5.1×10^3^ | -2.7% | 0 | 6 |
| MC4 | Model+CEH | Persistently low load | 2.4×10^2^ | -0.7% | 0 | 4 |
